# GenomeMatcher: A graphical user interface for DNA sequence comparison

**DOI:** 10.1186/1471-2105-9-376

**Published:** 2008-09-16

**Authors:** Yoshiyuki Ohtsubo, Wakako Ikeda-Ohtsubo, Yuji Nagata, Masataka Tsuda

**Affiliations:** 1 Department of Environmental Life Sciences, Graduate School of Life Sciences, Tohoku University, 2-1-1 Katahira, Sendai, 980-8577, Japan

## Abstract

**Background:**

The number of available genome sequences is increasing, and easy-to-use software that enables efficient comparative analysis is needed.

**Results:**

We developed GenomeMatcher, a stand-alone software package for Mac OS X. GenomeMatcher executes BLAST and MUMmer, and the detected similarities are displayed in two-dimensional and parallel views with similarity values indicated by color. Selection and re-computation of any subregions is easily performed and allows flexible and in-depth analysis. Furthermore, symbols for annotation data are displayed along the views, and the user can relate the genomic differences with annotation data. While bl2seq allows sub-Giga base comparison, three alignment programs, bl2seq, MAFFT and ClustalW, together with a dotmatch program allow comparative analysis of single-nucleotide level resolution. GenomeMatcher images can be saved as PDF and TIFF files for presentation. As examples of graphical ability of GenomeMatcher to show similarity in colors, we show two cases in *Burkholderia *and *Vivrio *strains that the nucleotide sequence of the second largest chromosome changes more rapidly than the largest chromosome.

**Conclusion:**

GenomeMatcher is efficient and easy-to-use stand-alone software for in-depth comparative analysis of two sequences. GenomeMatcher is useful for detecting similarities in DNA sequences ranging in size from a few to sub-Giga bases.

## Background

The number of available genomic sequences is growing rapidly, and comparisons among them has fruitful for identifying biologically and evolutionarily important traits. The tasks in comparative genomics include, (i) identification of conserved parts between two sequences, (ii) comparison of genomic structures, (iii) identification of sites of genomic rearrangement, (iv) identification of genomic islands, (v) by self-to-self comparison of a genomic sequence, identification of repetitive DNA sequences, which are often associated with IS elements, transposons, CRISPR (clustered regularly interspaced short palindromic repeats), integrons, origins of replication, transcriptional terminators, etc, and (vi) understanding those genomic features with respect to the annotated information. In addition, it is necessary to draw comparison images to present figures for presentation.

Several non-command-line tools have been developed that exhibit comparison graphics, including ACT [1], GATA [2], CGAT [3], ACGT [4], and G-InforBIO [5]. However, these existing graphic tools lack some of the following functions that would assist users in efficiently comparing two genomes in detail for the above tasks. First, the analysis scheme should allow the user to re-compute the similarity between any subregions of interest using different algorithms or parameters. The sensitivity with which similarities are identifyed depends strongly on these factors. When re-computability is not available, users cannot determine whether or not there are some similarities that the computation settings failed to identify. In addition, for efficient analysis, the re-computation process should require only limited handling of input devices, and in particular should not require the user to handle files or command lines, since these are often time-consuming. Second, in order to examine differences at nucleotide-level resolution, the exact start and end locations of a given matching pair as well as the base-to-base alignment of the pair should be readily obtainable. In addition, an analytical scheme that identifies sequence matches as short as few bases should be implemented in order to efficiently identify, for example, a terminal direct repeat of several base pairs relevant to the integration of a genomic island or an insertion sequence. The existing tools are not designed to identify sequence matches that are as small as, for example, two nucleotides. Third, annotation data should be easily referable in the process to examine the graphical results of similarities. Without this function, users cannot relate the differences to annotated information. Although some tools exhibit only locus tags present in the annotation file, users have to retrieve the relevant annotation for themselves, a time-consuming process. In addition, tools for comparative genomics should include a function to accept user-specified annotation data sets, so that the user can easily add genomic features they have identified, and view them in relation to pre-existing annotation data. Fourth, the similarity scores displayed graphically in colors should allow intuitive perception of similarities. Some tools exhibit sequence similarities by the thickness of a single color. However, the color resolution of the resulting images are lower than that of images drawn under a coloring rule that adopts various colors. Such a coloring rule should be modifiable in order to allow clearer recognition of the distribution of sequence similarities between given sequences. Lastly, not only a bitmap image but also a vector-formatted image of a comparison result should be provided in order to make it easy to prepare figures for presentation. Those tools that provide only bitmap images make it difficult for the user to modify generated images, for example in changing the color of a part of the image to stress some features or in removing unnecessary letters.

With these functions in mind, we developed GenomeMatcher, a graphical interface for existing programs (bl2seq[6,7], MUMmer [8], MAFFT [9] and ClustalW [10]), and provided for it with a tool named dotmatch that allows the detection of matches at lengths of a few nucleotides long. In GenomeMatcher, the re-computation of the sequence similarities of the specified sub-regions is possible, and sequence matches with few nucleotides long are detectable by dotmatch. User-specified annotation data sets are acceptable, and RGB(red, green, and blue)-colored comparison graphics, in which colors indicate similarities, can be saved as both vector-formatted images or bitmap images.

## Implementation

### Software arrangement

The GenomeMatcher system is programmed in Objective-C using the Cocoa framework with the associated XCode development tools (both by Apple Inc.). The inputs are DNA sequences in DDBJ/GenBank, single FASTA, and plain text formats. For sequences that have not yet been processed into the DDBJ or GenBank format, GenomeMatcher accepts annotations in a defined format (see online manual available at ). In GenomeMatcher, five comparison programs are used for sequence comparison: bl2seq, MUMmer, MAFFT, ClustalW, and a program for nucleotide level comparison (called 'dotmatch' here). The bl2seq and ClustalW are embedded in the application bundle, and users have to download MAFFT and MUMmer and specify the path in the setting window where these two programs are installed. The use of bl2seq and MUMmer is for the comparison of sequences longer than several hundred base pairs, while that of dotmatch and ClustalW is for sequences shorter than several thousand base pairs. MUMmer, MAFFT, and ClustalW are useful for obtaining an alignment of two sequences when the bl2seq program fails to align them due to insufficient similarities. In the dotmatch analysis, a pair of sequences is compared at nucleotide levels and all nucleotide matches longer than a specified length are displayed. The dotmatch analysis help identify repetitive sequence, which are often related to biologically important features. The seamless connection of these analytical methods allows the efficient identification of similarities in DNA sequences ranging in size from a few to sub-Giga bases.

## Results and discussion

### Rapid and efficient comparison by GenomeMatcher

**Figure 1:**
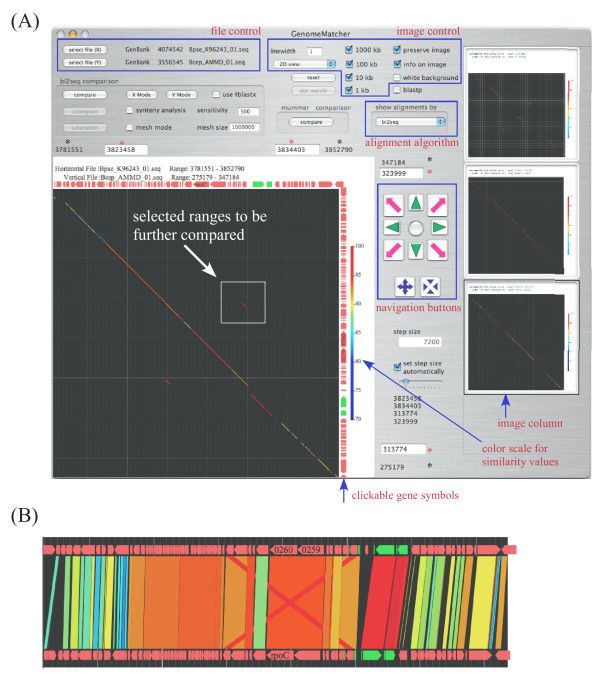
**Example of GenomeMatcher analysis**. Two sequences in the GenBank format are loaded and compared successively three times by bl2seq. Clicking the arrows besides the comparison image displays the text-based annotation information. **(A) **Clicking a stored image in the right image column converts it into a larger image. The black asterisks denote comparison ranges of the current image, and the red ones the ranges to be compared in the next analysis. **(B) **The representation of the comparison result of panel A in parallel view (parallel-flip-view, in which the upper sequence is flipped). See the instruction manual for more details.

Unlike other programs, GenomeMatcher does not require a pre-computed comparison result because it compares two sequences by running programs enclosed in the application bundle or placed on user's computer. When GenomeMatcher has already been launched and two DNA sequence files have been loaded, the user can obtain a comparative image just by starting the comparison function. When blastn is used, calculation and subsequent generation of a comparative image of typical bacterial genomes finishes in a few seconds on dual 3 GHz dual-core Xeon CPUs. The examples of analysis are shown in Fig. 1.

One characteristic feature of GenomeMathcer is its re-computability, and the user can repeatedly analyze the similarities between two DNA sequences using different parameters or different programs (blastn and tblastx of bl2seq or nucmer and promer of MUMmer). The programs tblastx and promer have more detection power than blastn and nucmer, respectively, thereby allowing the detection of genomic sub-regions that share low sequence similarities. The user can also obtain a text-based alignment of any subregions using bl2seq, nucmer, promer, MAFFT, and ClustalW.

The user can choose subregions by dragging and starting the re-computation. Such an operation allows the user to repeatedly gain a close-up view of a region of interest. As the length of the sequence displayed falls below a certain threshold (200 kb by default), gene symbols will appear along the comparison image, which upon clicking exhibits relevant annotation data. Thus, the user can relate the genomic differences to annotation data.

### Displaying similarities

GenomeMatcher displays blastn, tblastx, nucmer and promer comparison results in both linear and two-dimensional views. In the former view, sequences are placed in parallel to each other. In the latter view, two sequences are placed along the x- and y-axes, respectively, of a rectangular coordinate system. These two view modes have their own advantages, and users can choose either view depending on their purposes.

GenomeMatcher shows similarity values in colors that change gradually as the level of similarity changes (as similarity increases, dark blue changes to green, yellow, and red; the relation between similarity value and colour is displayed beside the comparative image). Therefore, the user can gain, at a glance, the information on which parts of the genome are more or less conserved. This contrasts with the existing programs, in which the intensity of the colour bands is proportional to the similarity [1] or monotonous [2,5].

The generated images can be saved as TIFF and PDF formats. The latter is more useful for the preparation of figures because PDF file images can be modified using commercially available graphic softwares.

### Mesh mode and catenation mode

Under an execution mode that we call mesh mode, the bl2seq program compares DNA sequences subregion by subregion and assembles the results to completion. The use of this mode is recommended for the following comparisons: i) long (>10 Mb) sequence comparison because comparison of a long sequence by bl2seq consumes a lot of memory and sometimes fails; and ii) comparison of very similar or identical sequences, because such comparison fails to identify some regions with considerable similarities.

**Figure 2:**
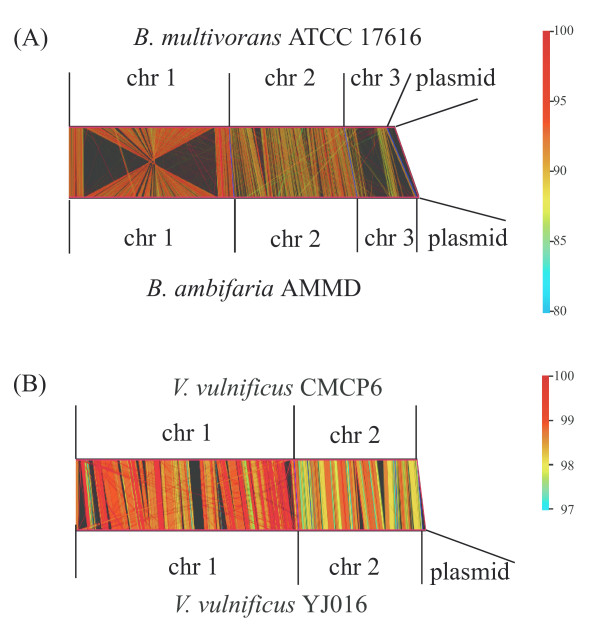
**Genome vs genome comparison**. Total genomic sequences were compared by catenation mode. Before the comparison, DNA sequences of all replicons within a genome were concatenated. As the compared sequences are circular entities, the starting nucleotide positions and direction of sequences were adjusted to improve graphics. Parallel drawings of comparison results are shown. chr; chromosome. **(A) *Burkholderia multivorans *ATCC 17616 vs *B. ambifaria *AMMD **Accession numbers for ATCC 17616 are AP009385–AP009388, Accession numbers for AMMD are CP000440–CP000443**(B) *Vibrio vulnificus *YJ016 vs *V. vulnificus *CMCP6 **Accession numbers for YJ016 are BA000037, BA000038, and AP005352. Accession numbers for CMCP6 are AE016795 and AE016796.

In another execution mode that we call catenation mode, the user can specify the individual sizes of the subregions, allowing, for example, replicon-by-replicon comparison of two multi-replicon genomes (Fig. 2AB). This mode also allows the user to compare concatenated genomes. For example, a sequence consisting of ten bacterial genomes was successfully compared to itself, thereby generating an image of reciprocal comparisons of 10 genomes in a single operation [see Additional file 1].

We here describe one of the scientific findings we found using GenomeMatcher's ability to show similarity values using colors. Fig. 2A shows the genome comparison result of two related *Burkholderia *strains having multiple replicons. As is clearly demonstrated by the colors, chromosomes 2 are less conserved than chromosomes 1. This is also the case in another set of related *Vivrio *strains (Fig. 2B). Although how this happens remains to be fully elucidated and much comparative analysis should be done before generalizing this finding, we propose that the second largest chromosomes evolve more rapidly than the largest chromosomes. One explanation for this is that the common ancestral replicon of the second largest chromosomes was introduced into the common ancestral cell, and at the moment of introduction, the nucleotide composition of the incoming replicon was significantly different from that of the replicon residing in the ancestral host cell. The higher divergence among the second largest chromosomes might be due to a higher speed of progression of amelioration on the alien composition of chromosome 2.

### X mode and Y mode

**Figure 3:**
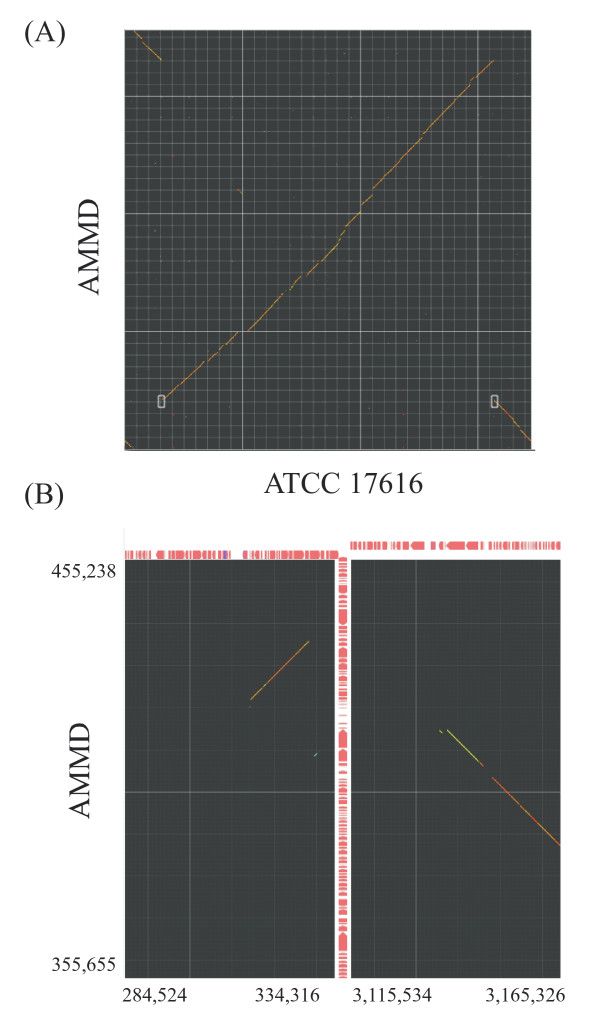
**Comparison of two distantly located areas**. The largest chromosome of *B. multivorans *ATCC 17616 was compared with that of *B. ambifaria *AMMD. (A) Starting X mode. Two white half-square rectangles indicate regions to be compared. (B) X-mode result of panel A. Exact comparison ranges are indicated.

In comparative genomic analysis, traces of large-scale genomic rearrangements and insertion of large genomic islands are often observed. To analyze the configuration of the boundaries of such events, the X mode and Y mode were implemented. In these modes, the comparative results of two horizontally or vertically separated areas are displayed in close context. Upon starting either mode, selection of an area of interest by dragging generates a half-square rectangle. By clicking the image, another half-square rectangle appears, and the user sets its location. The two selected areas are compared and displayed in another window (see Fig. 3 for an example of running X mode).

### dotmatch analysis

In most genomic comparison programs, the sequence similarities found are displayed. However, such programs cannot display detailed information on the gap regions where similarities are not detected. The dotmatch program in GenomeMatcher allows users to examine similarity levels in the gap regions by displaying sequence comparisons at the nucleotide level.

**Figure 4:**
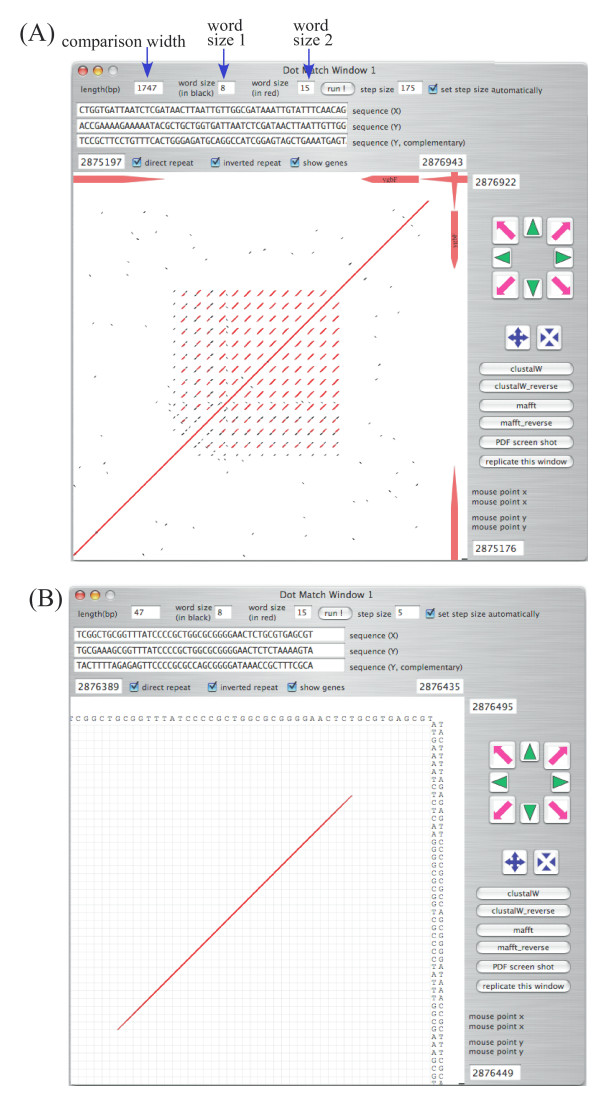
**Example of dotmatch analysis in GenomeMatcher**. (A) DNA regions of CRISPR [11] in *Escherichia coli *K12 MG1655 genome (U00096). MG1655 genome was placed both on x- and y axes. (B) Close-up view of panel A. Exact matches with sizes larger than 'word size 1' are shown in black and those larger than 'word size 2' in red. As in the main window, regions to be compared are easily be set by dragging or pressing the navigation buttons.

The dotmatch program implemented in GenomeMatcher is also useful for identifying repetitive sequences. Here we show, as an example, how a CRISPR is identified in dotmatch analysis (Fig. 4).

### Comparing long sequences

The unique functions of GenomeMatcher include BLAST comparison by both the mesh and catenation modes. These modes enable comparison, using an ordinary desktop or laptop computer, of very long sequences such as a pair of 300 Mb sequences that are larger than the largest chromosome of *Homo sapiens*.

### Merits and limits of two dimensional visualizations

The two-dimensional visualization of a result of the comparison of two sequences is an effective way to understand the commonalities and differences between two sequences. Especially in cases where genomic sequences are compared, this method could be essential, because huge text-based results are too hard to interpret. The GenomeMatcher software includes two types of two-dimensional representation of comparison results. One visualizes results from bl2seq or MUMmer, and the other visualizes perfect nucleotide matches at lengths as short as a few nucleotides (dotmatch). The merits of the former in both types of representation (see Fig. 1 panels A and B) include instant conversion of huge text-based results into two-dimensional representation. In addition, in both types of representation, the identity score of each HSP is reflected in the color of the corresponding line. The editable color scale of GenomeMatcher, starting from blue and changing gradually to green, yellow, and finally red, allows the user to perceive subtle differences in identity scores (for example see Fig. 2AB). No other tool to visualize genomic similarity developed to date provides higher color resolution than GenomeMatcher. For example, an approximately 1% difference in nucleotide identity is easily discernable in Fig. 2B. This color resolution is not possible to achieve for tools, in which identity scores are expressed as a thickness of a single color, as in ACT. A disadvantage of the visualization of bl2seq or MUMmer results includes that sequence similarities between relatively small tracts, which might be of biological importance, might be drawn enough small to be overlooked. However, this problem could be overcome, to some extent, by dotmatch, which makes it easy to identify very small nucleotide matches. For example, dotmatch often makes it easy to identify target site duplication of insertion sequences, which could be as small as two nucleotides long. On the other hand, dot-match analysis is not suitable for comparing long sequences, because as the sequence length increases the comparison image becomes too complicated to find important features.

### Merits and limits of GenomeMatcher

The merits of GenomeMatcher are the accessibility of annotations, the ease of use for non-computer-literate researchers, its fine graphical capability that allows users to perceive differences in similarity at a glance and even enables the generation of vector-formatted images, and the capability to compare a wide range of sequence lengths. Moreover, images generated by GenomeMatcher are not static, thus allowing consecutive dynamic analysis that is supported by the program's quick re-computability. The limits of the current version of GenomeMatcher are that it cannot compare three or more sequences at a time, as can ATC or MUMmer, and that it runs only on Mac OS X and not on other operating systems.

## Conclusion

GenomeMatcher is an easy-to-use interface for existing comparison programs that enables users to efficiently perform comparative genomics.

## Availability and requirements

Project name: GenomeMatcher project;

Project homepage: 

Operating systems: Macintosh OS X 10.3 or higher;

Programming language: Objective-C;

Any restrictions to use by non-academics: License needed for non-academic users.

## Authors' contributions

YO participated in the design, coding, and coordination of the study and drafted the manuscript and online manual. IW participated in the design of the software and improved usability and the online manual. YN and MT participated in the design and preparation of the manuscript. All authors read and approved the final manuscript.

## Supplementary Material

Additional file 1 Example of catenation mode: genomes vs genomes comparison. The genomic sequences of ten *Escherichia coli *strains were concatenated (51.2 Mb in total) and compared by using the catenation mode. The order of catenation is *E. coli *536, APEC O1, CFT073, UTI89, E24377A, HS, K12 MG1655, K-12 W3110, O157:H7 EDL933, and O157:H7 Sakai RIMD 0509952. To gain better resolution, the image width was set to 2,350 points. Blue and white lines, which are depicted automatically, indicate replicon and genomic boundaries, respectively. Click here for file
