# Supplementary figures and images for: GenomeMatcher: A graphical user interface for DNA sequence comparison
Source: BMC Bioinformatics. 2008 Sep 16;9:376. doi: 10.1186/1471-2105-9-376 (PMC2553346; doi:10.1186/1471-2105-9-376)

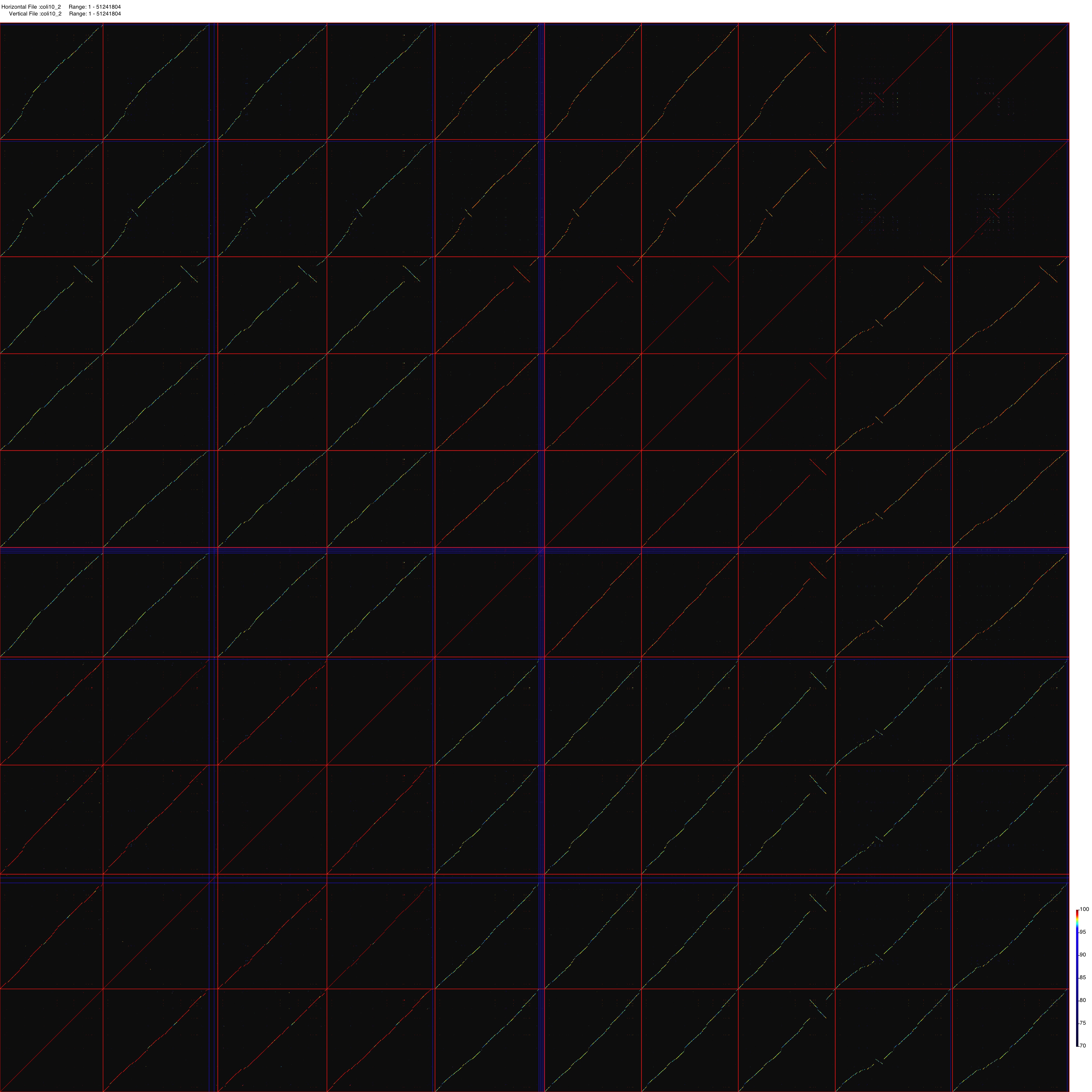

Supplement: Additional file 1 — Example of catenation mode: genomes vs genomes comparison. The genomic sequences of ten Escherichia coli strains were concatenated (51.2 Mb in total) and compared by using the catenation mode. The order of catenation is E. coli 536, APEC O1, CFT073, UTI89, E24377A, HS, K12 MG1655, K-12 W3110, O157:H7 EDL933, and O157:H7 Sakai RIMD 0509952. To gain better resolution, the image width was set to 2,350 points. Blue and white lines, which are depicted automatically, indicate replicon and genomic boundaries, respectively. [file 1471-2105-9-376-S1.jpeg]
